# Impact of advanced lithotripter technology on SWL success: ınsights from Modulith SLK ınline outcomes

**DOI:** 10.1007/s00345-025-05517-4

**Published:** 2025-02-25

**Authors:** Erhan Erdoğan, Gamze Şimşek, Alper Aşık, Göksu Sarıca, Kemal Sarıca

**Affiliations:** 1 Department of Urology, Sancaktepe Sehit Prof. Dr. Ilhan Varank Research and Training Hospital, Istanbul, Turkey; 2 Biruni University Medical School, Istanbul, Turkey; 3 Department of Urology, Medical School, Biruni University, Istanbul, Turkey

**Keywords:** Shock wave lithotripsy, Kidney stones, Modulith SLK inline, Treatment success, Lithotripter technology

## Abstract

**Aim:**

This study aims to evaluate the success rate of Shock Wave Lithotripsy (SWL) in treating kidney stones using the Modulith SLK Inline lithotripter, with a focus on the importance of device efficacy as emphasized in EAU guidelines.

**Patients and methods:**

This retrospective single-center study was conducted between June 2023 and June 2024. Inclusion criteria were adult patients (> 18 years) with radiologically confirmed renal stones smaller than 15 mm in diameter. Exclusion criteria included patients with solitary kidneys, significant renal functional deterioration, skeletal deformities, active urinary tract infections, pregnancy, or coagulopathies. Treatment outcomes were collected and analyzed in detail, considering patients’ demographic characteristics (age, gender) and stone parameters (size, location, and hardness [Hounsfield Unit, HU]). The SWL procedures were performed using the Modulith SLK Inline lithotripter (Storz Medical, Switzerland). The success of SWL was defined as achieving complete stone clearance or the presence of clinically insignificant residual fragments (CIRF) (< 4 mm). This study seeks to provide detailed insights into the optimal use cases of SWL as a non-invasive yet effective treatment option for smaller, more manageable stones.

**Results:**

The mean age of the 208 patients included in the study was 42.2 ± 12.7 years (18–75), with a male-to-female ratio of 1.9:1. The mean stone size across all patients was 10.3 mm, and the average HU value was 874.0 ± 283.2. Patients who achieved a completely stone-free status had significantly lower HU values (p = 0.049). The overall success rate of SWL was 78.8%, with 164 patients achieving complete stone clearance. When cases with clinically insignificant residual fragments (CIRF, < 4 mm) were included as successful outcomes, the overall success rate increased to 92.3%. This distinction highlights the inclusion of patients with small residual fragments that are deemed clinically irrelevant in the adjusted success rate. In cases with successful outcomes, the mean stone size was 10.3 mm, whereas it was 12.5 mm in patients with residual fragments or treatment failure. A statistically significant relationship was identified between stone size and treatment success rates (p < 0.001). In contrast, stone localization did not have a significant impact on SWL success rates (p = 0.377).

**Conclusions:**

SWL has demonstrated its effectiveness in kidney stone treatment with a 78.8% complete stone-free rate using the Modulith SLK Inline lithotripter. Higher success rates were achieved with smaller stones (< 15 mm) and lower HU values. These findings support the significance of advanced lithotripter technology in establishing SWL as a valuable non-invasive option for stones under 15 mm.

## Introduction

Urolithiasis is a significant health problem affecting millions of people worldwide. The incidence of kidney stones varies significantly across different regions of the world. The prevalence is reported to be approximately 10–15% in developed countries and continuing to increase, particularly in industrialized countries, due to dietary and lifestyle changes associated with modernization [1].

Based on the underlying risk factors, the disease may recur in a significant proportion of cases, potentially leading to upper urinary tract obstruction and infection-related complications that could result in irreversible damage if not managed promptly. Additionally severe renal colic may affect the life quality in these cases necessitating and urgent management at each attack.

Symptomatic and obstructing stones require rational management to achieve complete stone clearance with minimal complications and limited impact on the patient’s quality of life. Regarding the treatment, management of kidney stones have evolved from invasive surgical procedures to minimal invasive approaches over the last 3–4 decades. Currently, the management options for kidney stones include extracorporeal shock wave lithotripsy (SWL), flexible ureteroscopy (fURS), percutaneous nephrolithotomy (PCNL), and open surgery [2]. Additionally, recent studies have highlighted the applicability of robotic pyelolithotomy in specific cases, emphasizing its potential as an alternative for managing large kidney stones [3]. Based on the evidence based data published so far, available international guidelines (EAU-AUA) do offer SWL as the first option for renal stones sizing less than < 15 mm. [2, 4]. Each of these methods has specific indications (with certain advantages and disadvantages) based on stone (size, number, location, and hardness) as well as patient related factors. When evaluated with respect to the invasive nature of the alternatives, SWL remains as the only “true noninvasive” option applied for the management of kidney stones in all parts of the world [4].

SWL was first introduced in the early 1980s as a technique that uses shock waves generated outside the body to fragment urinary stones [5]. This method allows kidney stones to be disintegrated into smaller pieces, which then may pass spontaneously through the urinary tract [6]. Over the years, parallel to technological advancements noted lithotriptor technology, the safety and efficacy of SWL have improved considerably, making it a preferred method in the effective and safe management of both kidney or ureteral stones [7].

Despite its noninvasive, practical and highly safe application however, it has been well shown that the success rates of SWL applications may vary depending on several factors, such as stone size, stone location, hardness, and body mass index (BMI) of the cases treated [8]. Reported studies have shown that the success rates for stones smaller than 2 cm range between 70 and 90% [9]. However, the effectiveness decreases for larger or harder stones where repeated sessions and auxiliary procedures may be required.

On the other hand again, despite its widespread acceptance, SWL has some certain disadvantages such as the potential for renal injury in risk patients, the chance of incomplete stone clearance and the need for additional procedures in patients with residual fragments. However, due to its non-invasive nature, minimal anesthesia requirements, and the possibility of outpatient treatment, SWL remains a valuable option in the management of urolithiasis. Outcomes will be even more successful if the decision making is made with a very careful patient/stone selection based on the clear indications given in the published guidelines and the treatments are carried out with an effective lithotriptor [10].

This study focuses specifically on outcomes with the Modulith SLK Inline lithotripter, known for its precision in targeting and fragmenting stones. In line with EAU Guidelines for Urolithiasis treatment, successful SWL treatment relies on factors such as stone size, hardness, patient habitus, and the lithotripter used. This study aims to present data that demonstrates the success of this device when paired with guideline-based patient selection criteria. Thus, in this present study we aimed to evaluate the overall success rate of SWL performed with an effective lithotriptor in the non-invasive treatment of kidney stones in a single center.

## Patients and methods

This retrospective study was conducted between June 2023 and June 2024, following approval by the local Ethics Committee (approval number: 298/2024). Patients undergoing SWL for renal medium-sized (< 15 mm) stones were included in the study, and informed consent was obtained from all participants prior to the procedure.

While adult patients (> 18 years), with radiologically confirmed opaque renal stones (< 15 mm) located in the different parts of the kidney, were included in the study. Patients with a solitary kidney, renal functional deterioration, significant skeletal deformities, active urinary tract infections, pregnancy, or coagulopathies were excluded from the study program.

In addition to the plain abdominal film and sonographic evaluation, the diagnosis and detailed characteristics of renal stones were confirmed with non-contrast computed tomography (NCCT) in all cases. In addition to the location and size, the density of the stones was assessed in the NCCT images in terms of HU values.

All patients underwent a comprehensive clinical evaluation, including urinalysis, urine culture test, complete blood count, coagulation profile, and renal function tests. Patients were instructed to fast for at least 6 h prior to the procedure, and prophylactic antibiotics were administered in cases deemed necessary, such as patients with a history of urinary tract infections, immunosuppressive conditions, or signs of active infection, to minimize the risk of post-procedural complications.

SWL procedures were performed using a Modulith SLK inline lithotripter (Storz Medical, Switzerland), under analgesic treatment with non-steroidal anti-inflammatory drugs (NSAIDs). Patients were positioned in the supine position, and stones were localized using fluoroscopy or ultrasonography. Regarding stone localization during treatment, the inline imaging capabilities of the MODULITH SLK Inline lithotripter system increase precision by allowing real-time visualization of stone targeting and highly effective fragmentation. This not only improves stone clearance rates, but also reduces the risk of complications. The unique stone-directed shockwave delivery with perfect alignment (keeping the stone always in the shockwave path) offers, an innovative and patient-centered approach to SWL, providing a non-invasive yet highly effective treatment solution for stones. Sedative analgesia with pethidine was administered in selected cases, but none of the patients required general anesthesia. A total of 3000 shock waves were delivered per session.

Treatment sessions were repeated at 7-day intervals, with a maximum of three sessions per patient, depending on the degree of stone fragmentation and the patient’s response to treatment.

To assess the stone-free (SF) status, a plain abdominal X-ray (KUB) was performed on the 7th day after the last SWL session. Final stone-free status was evaluated with non-contrast CT (NCCT) three months after the last treatment session. Complete stone clearance (stone free status) was defined as the absence of any detectable stones or the presence of CIRF (less than 4 mm).

Failure was defined as the presence of residual stone fragments larger than 4 mm after three successful SWL sessions. Symptomatic patients was offered alternative treatment options such as ureteroscopy (URS) or percutaneous nephrolithotomy (PCNL), depending on the size and location of the residual stones.

For statistical analysis, mean and standard deviation (SD) values were calculated. Independent t-test and Mann–Whitney U test were used for this purpose. The Chi-square test was employed for categorical parameters. One-way ANOVA was used to compare stone-free rates across different stone localizations. A *p*-value of less than 0.05 was considered statistically significant.

## Results

A total of 208 patients undergoing SWL were included in this study. The mean age of the patients was 42.2 ± 12.7 years, with an age range of 18–75 years. Among these patients, 137 (65.9%) were male and 71 (34.1%) were female, with a male-to-female ratio of 1.9:1. Baseline demographic characteristics are presented in (Table 1).

**Table 1:** The demographic characteristics of the patients

Demographic characteristics	Value
Patients (n)	208
Mean age (years ± SD)	42.2 ± 12.7
Male patients (n, %)	137 (65.9)
Female patients (n, %)	71 (34.1)

When analyzing treatment success, there was no statistically significant relationship between gender and treatment outcomes (*p* = 0.60).

The distribution of stones based on their localization within the kidney and corresponding treatment outcomes are summarized in (Table 2).

**Table 2:** Stone localization distribution and treatment success rates and outcomes for each localization

Stone localization	Number of patients (n)	Percentage (%)	Stone-free (n, %)	Residual fragments (n, %)	Failed (n, %)
Renal pelvis	58	27.9	49 (84.5%)	5 (8.6%)	4 (6.9%)
Upper calyx	27	13.0	23 (85.2%)	1 (3.7%)	3 (11.1%)
Middle calyx	56	26.9	43 (76.8%)	9 (16.1%)	4 (7.1%)
Lower calyx	67	32.2	49 (73.1%)	13 (19.4%)	5 (7.5%)
Total	208	100	164 (78.8%)	28 (13.5%)	16 (7.7%)

The chi-square test evaluating the relationship between stone localization within the kidney and treatment success rates indicated no statistically significant difference between stone localization and treatment success (*p* = 0.377). The overall evaluation of success rates for all stones, SWL was found to be successful in 164 out of 208 patients (78.8%) with completely stone-free status, residual fragments (< 4 mm) were present in 28 patients (13.5%), and treatment failure occurred in 16 patients (7.7%) (Table 2). Based on these outcomes we may say that the overall success rate of SWL in renal stones sizing < 15 mm will be 92.3% with the inclusion of asymptomatic small fragments (< 4 mm) which could be passed spontaneously over time.

The mean HU of the stones in the patients included in the study was determined to be 874.0 ± 283.2 (263–1781). While this value was 862.7 ± 286.2 in patients who achieved a completely stone-free status, it was 1032.5 ± 360.2 in those with residual fragments or treatment failure, statistical analysis of HU difference between these groups showed a statistically significant difference (*p* = 0.049). It was particularly noted that stones with an HU value below 1000 had a higher likelihood of being effectively fragmented and cleared with SWL.

When examining the relationship between stone size and treatment success, while the mean stone size was calculated as 10.3 mm (7.4–15.2 mm) in patients with successful outcomes, it was 12.5 mm (9.1–13.9 mm) in those with residual fragments or treatment failure. Again a statistically significant relationship was found between stone size and SWL success (*p* < 0.001).

Finally, the evaluation of complications observed in our study group is summarized in Table 3, which also includes additional analysis of their relationship with stone size and location. Hematuria was identified as the most common complication, occurring in 132 patients (63.4%). A statistically significant relationship was found between hematuria and stone size, with a higher incidence observed in patients with stones ≥ 10 mm (*p* = 0.045). Although hematuria showed a trend towards being associated with stone location, this did not reach statistical significance (*p* = 0.082). Hematuria was managed conservatively with high fluid intake and resolved spontaneously in the vast majority of cases.

**Table 3:** Complications of SWL categorized by stone size and location

Complication	Frequency (n, %)	Stone size (< 10 mm vs. ≥ 10 mm) *p*	Location *p*
Hematuria	132 (63.4)	0.045	0.082
Renal colic	11 (5.3)	0.067	0.110
Steinstrasse	1 (0.5)	–	–
Renal hematoma	2 (0.9)	0.032	0.050

Renal colic was the second most common complication, observed in 11 patients (5.3%). The occurrence of renal colic was not significantly associated with stone size (*p* = 0.067) or location (*p* = 0.110). Symptoms were successfully managed with analgesic and antispasmodic agents in all affected patients.

Steinstrasse was noted in only one patient (0.5%), who was successfully treated with ureteral stent placement.

Lastly, renal hematoma formation was observed in two patients (0.9%). A significant relationship was found between renal hematoma and both stone size (*p* = 0.032) and location (*p* = 0.050), with stones located in the lower calyx being associated with a higher risk of hematoma formation. This suggests that larger stones and specific anatomical factors, such as lower calyceal location, may contribute to the increased risk of hematoma formation.

## Discussion

The EAU Guidelines for Urolithiasis treatment emphasize that the success of SWL is influenced by factors including stone characteristics, patient habitus, and the efficacy of the lithotripter itself. This study underscores the importance of utilizing advanced lithotripter technology, such as the Modulith SLK Inline, which incorporates features designed to optimize treatment outcomes. The precision of its real-time imaging capabilities and its ability to deliver targeted shock waves may enhance the efficiency of stone fragmentation and clearance. While this study does not provide a direct comparison with other devices, the observed outcomes suggest that the technological advancements in the Modulith SLK Inline play a significant role in achieving high success rates in SWL.

Poor lithotripter performance or improper case selection often results in suboptimal outcomes and higher retreatment rates. In contrast, advanced shockwave technology like the Modulith SLK Inline, combined with careful patient selection per EAU criteria, provides a pathway to high success rates in SWL without resorting to invasive procedures. This study’s findings validate the importance of choosing an effective lithotripter and adhering to best practices, ultimately supporting the Modulith SLK Inline’s role in improving patient outcomes and minimizing the need for surgical alternatives.

SWL has been clinically introduced in 1980 by Chaussy et al., and became the preferred treatment modality in the “true noninvasive” management of urinary stones with the safe and effective results obtained both in adults and children [5]. The practical and cost effective characteristics of this modality have led the urologists to perform it in more than 90% of the medium sized (10–20 mm) stones in adult patients without any anesthesia requirement as well as hospitalization [2, 4]. Currently both EAU and AUA guidelines still recommend this particular modality as the preferred option for stones sizing less than 20 mm [2, 4, 11].

In addition to these facts, as we all know well, the clinical practice patterns of urinary stone management have been found to be affected to a certain extent by the COVID-19 pandemic. Unprecedented introduction of COVID-19 has dramatically influenced all parts of medicine and changed our practice patterns in stone management to a certain extent by selecting the best approach particularly in urgent cases and postpone and/or reschedule elective procedures to limit the risk of infection spread. Based on the facts and evident changes in the practice patterns, elective stone procedures such as RIRS and PCNL needed to be postponed during the outbreak of the pandemic. While timely management of these cases in the emergency department was crucial, other urgent solutions including medical management to some extent and widespread application of SWL (in an outpatient based manner) gained more importance. SWL was the only treatment choice which allowed stone management without any anesthesia and relevant risks for the spread of the infection during this critical period.

Significant compulsory alterations in stone related interventions were noted in all parts of the world and in a well conducted survey during this period, vast majority of the experts (89.4%) tended to change their treatment strategy particularly in patients referring to the emergency departments during COVID-19 period. As a “true noninvasive” modality, SWL emerged as a very valuable alternative during this highly critical era (in other words its revival has been realized) with advantages of no anesthesia requirement, chance of treatment away from the patient and less risk of disease spread [12, 13].

However, despite its noninvasive, practical and successful natüre, SWL has certain disadvantages. As a major factor, it is well known that the efficacy of this modality tends to diminish in patients with hard (with high density) and larger sized stones (> 20 mm). Some minor complications (pain, hematuria and obstruction) and auxiliary procedures for complete stone clearance could be required in certain percent of such cases [1]. Thus, treatment outcomes may be influenced by some unpredictable stone (size, composition, location) and patient (BMI, collecting system anatomy) factors. A dedicated experienced team, careful patient selection along with a cooperative work with the cases are crucial factors for a successful SWL procedure. [2, 8, 14–18]. Last but not least, certain stone types, such as cysteine or calcium oxalate monohydrate stones, may not respond optimally to SWL and may necessitate alternative treatment methods [19].

In the light of the facts mentioned above, it is clear that although the role of minimal invasive flexible ureteroscopic stone treatment tended to increase over the last 2–3 decades, accumulated published data in literature has revealed comparable stone free rates between these two modalities particularly for small (less than 1 cm) and medium (1–2 cm) sized renal stones [20, 21]. However, it should be kept in mind well that, despite its minimally invasive nature, ureteroscopic procedures may reveal certain complications as a major disadvantage when compared with SWL [22, 23].

The reported success rates after SWL in terms of SF status were found to range between 48 and 85%. Related to this issue, while Padhye et al. reported an overall stone-free rate of 91.7% in upper tract stones [24], Ghimire et al. reported a clearance rate of 91.1% in 112 patients with renal stones [25]. Additionally, success rates of 74% for kidney stones and 88% for ureteric stones were reported by Al-Marhoon et al. [26]. When the outcomes were evaluated in a stone size based manner, Gupta et al. found a higher stone clearance rate of 90.8% for relatively smaller stones (< 11 mm) with a mean stone density value of 750 HU [27]. These results were inconsistent with the outcomes reported by Hamal et al., where 85.9%, 90.25%, and 50.5% success rates for the upper, middle, and lower calyx, respectively [28].

With respect to the complications associated with SWL applications, limited minor (flank region pain or discomfort and microscopic haematuria) complications have been reported in the literature [22, 23, 29].

Evaluation of our results revealed that the overall complete SF rate after SWL treatment was found to be 78.8%. Evaluation of the success rates on stone location based manner, revealed that while a complete SF status was achieved in 84.5% of renal pelvic stones these rates were 85.2% in cases with upper calyx stones,76.8% in the middle and 73.1% in lower calyx stones. In addition to the size of the stone the hardness (HU value) was found to be another critical parameter to be taken into account for a successful outcome after SWL. Related to this issue, a statistically significant difference in HU values was observed between the groups. The mean HU was 862.7 ± 286.2 in patients with successful outcomes, while it was 1032.5 ± 360.2 in those with residual fragments (mean difference = 169.8 ± 74.0, *p* = 0.049), indicating that higher HU values are associated with unsuccessful outcomes.

Last but not least, stone size was calculated as 10.3 mm in patients with successful outcomes, while it was 12.5 mm in those with residual fragments or treatment failure. A statistically significant relationship was found between stone size and SWL success (*p* < 0.001).

In the light of our findings and the reported data in the literature we may definitely emphasize the important place of SWL both in adults and pediatric cases when indications are selected in a proper way. SWL has gained more importance particularly after the critical COVID 19 era and it is still the best treatment alternative in the noninvasive management of upper tract calculi with its high success and significantly limited complication rates. The application needs to be done by an experienced team based on technical requirements. Patient selection is the key factor and as our results also indicate it seems to be the preferred treatment choice for stones sizing less than 15 mm with a HU value of < 1000. By this way the vast majority of such stones will be treated in a safe manner without any need for more invasive procedures which are significantly prone to more severe complications. Patients need to be a part of the decision making period where they really will get detailed information about the advantages and disadvantages of all available management options for a rational decision.

Our study is not free of limitations. First of all, the retrospective natüre of the methodology and relatively smaller number of the cases (although it is an acceptable caseload treated in a year period) might constitute a major drawback. Additionally, lack of long term evaluation of the cases for possible complications might be another limitation to be stated. However, as our main aim is to outline the efficacy and safety of SWL in the management of carefully selected cases, we believe that our results will highlight the valuable place of this modality in the “true noninvasive” management of such cases. SWL has its important place among the other treatment alternatives particularly demonstrating its “revival” after COVID-19 era with guidelines based on clear indications.

## Conclusions

In light of our findings and existing literature, SWL plays a crucial role in the non-invasive management of small to medium-sized stones in adult patients. SWL’s significance has grown, especially following the COVID-19 era, positioning it as a preferred treatment alternative for upper urinary tract stones due to its high success rate and minimal complications when performed by an experienced team according to guideline-based indications. Our study demonstrated that SWL, using the advanced Modulith SLK Inline lithotripter, achieved a complete stone-free rate of 78.8%. However, as stone size and hardness increased, success rates declined, emphasizing the importance of precise case selection. Patients with smaller stones (< 15 mm) and lower HU values achieved notably higher success rates. These findings reinforce that SWL, supported by advancements in lithotripter technology, remains a valuable non-invasive option for stones under 15 mm. The enhanced capabilities of the Modulith SLK Inline lithotripter highlight its effectiveness in optimizing treatment outcomes and reducing the need for surgical interventions.

## Data Availability

No datasets were generated or analysed during the current study.
